# Blood Group Type and COVID-19 Severity at Tertiary Care Centre, Nepal: An Observational Study

**DOI:** 10.31729/jnma.9106

**Published:** 2025-07-01

**Authors:** Nabin Kumar Rauniyar, Late Shyam Pujari, Rojina Manandhar

**Affiliations:** 1 Department of Internal Medicine, Nepal Police Hospital, Kathmandu, Nepal; 2 Department of Obsteric/ Gynaecology, Nepal Police Hospital, Kathmandu, Nepal

**Keywords:** *blood group*, *SARS-CoV-2*, *disease severity*, *comorbidities*

## Abstract

**Introduction:**

The respiratory infection with COVID-19 caused by the novel coronavirus SARS-CoV-2, emerged in December 2019 and was later declared a global pandemic by the world health organization. Clinical observations suggest the role of ABO blood types in susceptibility to COVID-19 infection. The study was designed to find the proportion of blood group distribution across the severity of COVID-19 patients.

**Methods:**

This is an observational cross-section study conducted at a tertiary care hospital. All COVID-19 patient visiting hospital within January to December 2021 were included in the study. Their demographic profile, blood group and COVID-19 severity were recorded. Descriptive analysis of the date was done.

**Results:**

There were 399 confirmed case of COVID-19 during study period, out of which 278 (69.7%) were male and 198 (49.62%) were aged between 21-40 years. There were 78 (31.45%) amongst mild COVID-19 and 32 (39.04%) amongst severe COVID-19 (Bilateral Pneumonia) having A positive blood group.

**Conclusions:**

The study suggests that patients with blood group A positive were more in the mild COVID-19, severe COVID-19 group.

## INTRODUCTION

The respiratory infection with COVID-19, caused by SARS-CoV-2, presents with a wide spectrum of symptoms ranging from mild illness to critical respiratory distress.^1-3^ Several host factors such as age, gender, and comorbidities influence disease severity. Among these, the ABO blood group system has gained attention, with studies suggesting that certain blood types may affect susceptibility and outcomes in COVID-19.^4-6^

Previous research indicates that blood group A may be associated with increased severity, while group O might offer a protective effect.^4,5,8^ However, results have been inconsistent and population-specific. There is limited data from Nepal on this topic^7,8^, which necessitates local investigation. Therefore, this study was designed to find the proportion of blood group distribution across the severity of COVID-19 patients.

## METHODS

This is an observational cross-section study conducted at Kantipur Hospital, Tinkune, Kathmandu which is a Tertiary Care hospital. The study was done on all patients with confirmed COVID-19 and known blood groups, with retrospective data from January to December 2021, Ethical clearance was taken from ethical review board of Nepal Health Research Council (NHRC) (Reference number: 1763). The accessible populations were record of all COVID-19 positive patients visiting outpatient department or admitted to inpatient during the study period. The inclusion criteria required patients older than 18 years of age, tested positive for SARS-CoV-2 by RT-PCR. Incomplete data were excluded from the study. The data included for analysis were age, gender, blood group, and severity of COVID-19. We accessed records of all COVID-19 positive patients attending outpatient or inpatient services. Extracted variables included: Demographics: age, sex, religious affiliation, Clinical severity: categorized as asymptomatic; mild URTI; moderate LRTI; severe bilateral pneumonia; ARDS; or mortality, Primary outcomes: oxygen therapy (yes/no), non- invasive ventilation (NIV), and invasive mechanical ventilation.

Blood group testing is inexpensive, widely available, and easily integrated into routine care, making it a practical parameter for large-scale epidemiological analysis. Utilizing existing hospital or personal records that include blood group data allows for efficient research without the need for additional financial or logistical burden.

The severity of COVID-19 was on the record were Asymptomatic, Mild (Upper Respiratory Tract Infection), Moderate (Lower Respiratory Tract Infection), Severe COVID-19 (Bilateral Pneumonia), Severe COVID-19 (Acute Respiratory Distress Syndrome), Mortality. The required data were extracted from the record and saved in MS Excel 2018 for analysis. A descriptive analysis was done in excel sheet to calculate proportion of various blood group, severity of COVID-19 patient oxygen requirements and use of noninvasive ventilation (NIV). Data was then transferred to csv format and Python 3.13.5 was used to study relationship between blood group and severity of COVID-19 using heat map.

## RESULTS

There were a total of 399 patients included in the study out of which 278 (69.67%) were male, and 121 (30.32%) were female. There were 388 (97.24%) Hindu, 9(2.25%) Buddhists and 2 (0.50%) Muslims participants. There were 15 (3.75%) in ≤20 years, 198 (49.62%) in 21-40 years age range and 120 (30.07%) in the 41-60 years group, 60 (15.04%) in 61-80 years and 6 (1.5%) in 81 years and above. Amongst the total patient A positive patients were 133 (33.34%), (Table 1).

There were 248 (62.16%) patients with mild COVID-19 and 2 (0.50%) patient had mortality amongst the total population considered for the study (Table 2).

**Table 1:** Blood group amongst patient with COVID-19 positive patients (n=399).

Blood Group	n(%)
A Positive	133(33.34)
AB Positive	45(11.28)
B Positive	101(25.31)
A Negative	4(1.00)
B Negative	6(1.5)
O Positive	104(26.06)
O Negative	6(1.50)

**Table 2:** COVID-19 severity amongst the patient included in the study (n=399).

Severity from the COVID-19	n(%)
Asymptomatic Covid	20(5.01)
Mild Covid (URTI)	248(62.16)
Moderate Covid (LRTI)	42(10.53)
Severe Covid (B/L pneumonia)	85(21.30)
Severe Covid (ARDS)	2(0.50)
Mortality	2(0.50)

URTI: Upper Respiratory Track Respiratory Track Infection;B/L: respiratory Distress Syndrome infection; LRTI: Lower Bilateral; ARDS: Acute

**Table 3:** Oxygen requirement amongst various blood group type in COVID-19 patient (n=399).

Oxygen	Blood Group							Total
	A +v	AB +ve	B +ve	A -ve	B -ve	O +ve	O -ve	
Yes n(%)	36(37.11)	15(15.46)	23(23.71)	0	1(1.03)	21(21.65)	1(1.03)	97(24.31)
No n(%)	97(31.12)	30(9.93)	78(25.83)	4(1.32)	5(1.65)	83(27.48)	5(1.65)	302(72.69)
Total n(%)	133(34.34%)	45(11.28)	101(25.31)	4(1.01)	6(1.50)	104(26.7)	6(1.50)	399(100)

A +ve=A positive; B + ve=B positive; A - ve=A negative; B - ve=B negative; AB + ve=AB positive;, O + ve= O positive; O - ve= O negative

**Figure 1:**
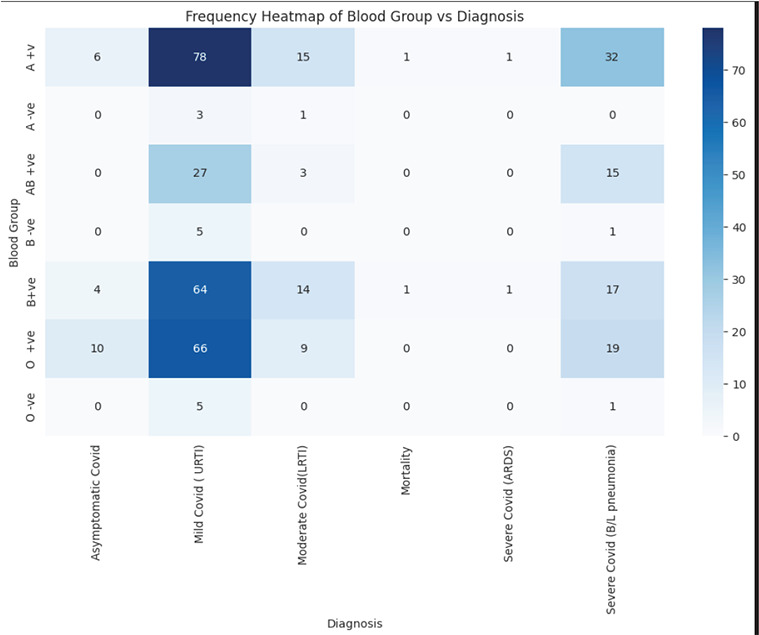
Heat map showing relationship between Blood Group and COVID-19 severity (n=399). A +ve=A positive; B + ve=B positive; A - ve=A negative; B - ve=B negative; AB + ve=AB positive; O + ve= O positive; O - ve= O negative

There were 78 (31.45%) amongst mild COVID-19 and 32 (39.04%) amongst severe COVID-19 (Bilateral Pneumonia) having A positive blood group (Figure 1)

Oxygen was required in 97 (24.31%) patients amongst whom oxygen requirement in A+ was 36 (37.11%), (Table 3) Amongst patient requiring oxygen 16 patients (4.01%) required non-invasive ventilation (NIV) which included 8 (50%) A+ patient, 5 (31.25%) B+, and 3 (18.75%) O+ patients. Invasive mechanical ventilation was required in 2 patients (0.5%), one each from the A+ and B+ blood groups.

**Figure 2:**
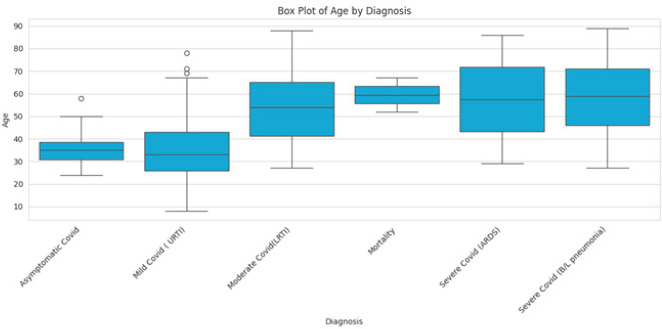
Boxplot showing relationship between age and severity of COVID-19 (n=399).

Among the total 399 COVID-19 patients, there were 2 deaths (0.5%), both occurring in the age group of 4180 years (Figure 2).

## DISCUSSION

In this study, patients with A positive blood group showed a notable presence in both mild and severe categories of COVID-19. A total of 78 (31.45%) individuals with A positive blood group were categorized under mild illness, and 32 (39.04%) experienced severe disease with bilateral pneumonia. Two deaths were recorded, one in a patient with A positive and another with B positive blood group. These patterns suggest that A positive might be related to greater disease severity in COVID-19 infection. The distribution indicates that individuals with A+ and B+ blood groups tended to experience more severe illness, while O+ cases were present across severity levels but without any recorded mortality.

Such a pattern highlights the potential role of ABO blood group antigens in modulating host susceptibility and disease progression, possibly through interactions with the immune system or viral entry mechanisms. These trends may reflect underlying immunological differences, such as anti-A antibody presence in non-A blood groups and pro-inflammatory responses mediated by blood group-specific antigens.

This observation aligns with previous reports by Zhao et al.^4^ and Chen et al.^3^, both of whom found blood group A associated with higher susceptibility, and blood group O possibly offering a protective role. The association is further supported by Golinelli et al. ^5^, who reported similar trends in their meta-analysis. However, unlike the findings by Padhi et al.^14^ in India, where B+ was linked to higher mortality, our study observed no deaths in B+ patients despite their high frequency, suggesting that ABO-COVID-19 associations may vary by population.

Severe cases were more frequent in A+, B+, and O+ groups, with both deaths occurring in A+ and B+ patients, possibly indicating increased vulnerability. This may be explained by higher binding affinity of SARS-CoV-2 to ACE2 receptors in A blood group individuals, as proposed by Gurung et al. ^8^ and supported by biological studies that suggest the presence of A antigens may facilitate stronger receptor interactions ^19^. In contrast, no mortality was recorded among O+ cases in our cohort, which supports earlier findings by Eatz et al.^15^, reinforcing the proposed protective role of O group individuals. Genetic analyses by Ellinghaus et al. ^16^ further provide evidence that ABO blood group loci are linked to variations in inflammatory response, contributing to differential severity among groups. Ellinghaus et al.^15^ identified loci on chromosome 3p21.31 and 9q34.2 (ABO locus) associated with severe COVID-19, suggesting that not just the blood group antigens but their genetic regulatory regions may influence cytokine levels and lung injury outcomes. This genetic insight supports the idea that blood group- related genetic polymorphisms can modulate immune responses and may partly explain the variability in clinical manifestations among different individuals.

Respiratory complications were also more prominent in blood group A patients. Oxygen therapy was required in 97 (24.31%) patients. Of these, 36 (37.11%) were A+, 23 (23.71%) were B+, and 21 (21.65%) were O+. Non-invasive ventilation (NIV) was needed in 16 (4.01%) patients, with 8 (50%) being A+, 5(31.25%) B+, and 3(18.75%) O+. Two patients, one A+ and one B+, required invasive mechanical ventilation.

This further supports the role of blood group in respiratory complications, aligning with mechanisms proposed by Guillon et al.^13^, where anti-A antibodies may inhibit viral entry in non-A blood groups. These findings are consistent with earlier reports by Zhao et al. ^4^, Chen et al. ^3^, and Golinelli et al. ^5^, which associated blood group A with increased risk and group O with a possible protective role. Biological mechanisms may involve greater binding affinity of SARS-CoV-2 to ACE2 receptors in A+ individuals, as proposed by Gurung et al.^8^ and supported by mechanistic models from Yamamoto et al. ^19^. Genetic studies by Ellinghaus et al.^16^ further suggest the involvement of loci that influence inflammatory responses, providing an explanation for variable outcomes by blood type. Furthermore, Guillon et al.^13^ demonstrated that the SARS-CoV spike protein's interaction with ACE2 could be inhibited by anti-A antibodies, suggesting a direct antiviral role for naturally occurring antibodies in non-A individuals a mechanism likely mirrored in SARS-CoV-2. This suggests that blood group-related antibodies may have a protective effect beyond preventing viral entry, possibly by modulating early immune activation and inflammatory pathways.

In this study, B+ blood group showed high frequency of severe illness but no deaths. This contrasts with the report by Padhi et al.^14^ in India, where B+ was associated with high mortality. A study from Pakistan by Shah et al.^20^ also found a strong association between B+ and severe disease, showing variation in regional patterns. No deaths were reported in patients with O+ blood type, supporting earlier observations by Eatz et al.^15^ regarding its protective nature. In contrast, the study by Elawamy et al.^18^ conducted in Libya found no significant correlation between ABO blood group and COVID-19 outcomes, emphasizing the need to consider local demographic, clinical, and genetic differences. Modeling studies by Miotto et al. ^17^ propose that transmission dynamics influenced by ABO type could shape disease patterns, suggesting that blood group profiling may help guide public health strategies during outbreaks. Miotto et al.^17^ employed agent-based models to simulate population-level dynamics and observed that blood group distribution may impact overall virus transmission, reinforcing the relevance of ABO-based risk stratification not only at an individual but at a societal level. These observations imply that public health measures could be tailored to population blood group distributions to mitigate the impact of future infectious outbreaks.

Our study underscores the importance of targeted clinical vigilance for patients with A+ blood types, especially those with underlying health conditions. Although findings are consistent with national ^8^ and international data ^4,5,9-11^, further multicentric and genetic studies are warranted to unravel the complex interaction between host blood type, immune response, and SARS-CoV-2 pathogenicity.

Given the routine nature of blood group testing in clinical settings and its cost-effectiveness in a resource- limited country like Nepal, this parameter may hold value as a supplementary marker in triaging or risk- stratifying COVID-19 cases. Since blood grouping is part of standard hospital admission and OPD records, its use in observational studies is logistically feasible and requires no additional resource investment.

However, several limitations should be acknowledged. This was a single-center retrospective study, and the data relied on pre-existing hospital records. Comorbidities such as COPD, hypertension, diabetes, and smoking status were observed to be associated with severe outcomes, but these were not controlled for through multivariate analysis. The small number of patients needing mechanical ventilation or experiencing mortality limits the statistical power to draw firm conclusions regarding death risk by blood group. Moreover, genetic polymorphisms and laboratory markers of inflammation were not included in the analysis, which could have provided a deeper understanding of the observed patterns.

Despite these limitations, the study provides useful insights and highlights a potential link between blood group and COVID-19 severity in the Nepali population. Future research should include multicentric collaboration, prospective study designs, and integration of genetic and inflammatory biomarkers. Such efforts would allow more robust and generalizable conclusions, especially for guiding health policies and clinical strategies in similar low-resource settings.

In conclusion, our study adds to the growing body of evidence that blood group A positive may be associated with increased severity of COVID-19. While group O appears to exhibit a lower risk for critical outcomes, more research is needed to clarify the biological underpinnings of this trend. In the context of Nepal's healthcare system, incorporating blood group information into triage and patient monitoring strategies may improve efficiency, especially during periods of healthcare system strain such as pandemics.

## CONCLUSIONS

This study highlights the possibility of A positive to be related with the severity of COVID-19 which was consistent with other findings. Blood group A positive were more in the mild COVID-19, severe COVID-19 group.
